# Vitamin D and Pain: Vitamin D and Its Role in the Aetiology and Maintenance of Chronic Pain States and Associated Comorbidities

**DOI:** 10.1155/2015/904967

**Published:** 2015-04-19

**Authors:** Edward A. Shipton, Elspeth E. Shipton

**Affiliations:** Department of Anaesthesia, University of Otago, Christchurch, Corner of Riccarton and Hagley Avenues, Christchurch 8042, New Zealand

## Abstract

The emergence of new data suggests that the benefits of Vitamin D extend beyond healthy bones. This paper looks at Vitamin D and its role in the aetiology and maintenance of chronic pain states and associated comorbidities. The interfaces between pain and Vitamin D and the mechanisms of action of Vitamin D on pain processes are explored. Finally the association between Vitamin D and pain comorbidities such as sleep and depression is investigated. The paper shows that Vitamin D exerts anatomic, hormonal, neurological, and immunological influences on pain manifestation, thereby playing a role in the aetiology and maintenance of chronic pain states and associated comorbidities. More research is necessary to determine whether Vitamin D is useful in the treatment of various pain conditions and whether or not the effect is limited to patients who are deficient in Vitamin D.

## 1. Introduction

A recent surge of published data on the proven or potential effects of Vitamin D has raised much interest in the medical community. The primary role of Vitamin D is the regulation of serum calcium levels within a narrow range. Vitamin D plays an essential role in bone formation, maintenance, and remodelling, as well as in muscle function. However, the emergence of new data suggests that the benefits of Vitamin D extend beyond healthy bones. Of great interest is the role it could play in optimising neuromuscular functioning, reducing inflammation, and decreasing the risk of many chronic illnesses; these include a variety of cancers, autoimmune diseases, infectious diseases, and cardiovascular diseases [1–5]. Research has shown that Vitamin D exerts anatomic, hormonal, neurological, and immunological influences on pain manifestation, thereby playing a role in the aetiology and maintenance of chronic pain states and associated comorbidity [1, 6–8].

## 2. Vitamin D

### 2.1. Synthesis, Absorption, and Metabolism of Vitamin D

Chemically, Vitamin D is a fat soluble secosteroid (i.e., a steroid in which one of the bonds in the steroid rings is broken). There are various forms of Vitamin D; the most common forms are Vitamin D_3_ (cholecalciferol) and Vitamin D_2_ (ergocalciferol). Both are collectively known as calciferol. Although it is called a vitamin, Vitamin D is really a hormone as it can be produced endogenously by humans. In the skin, 7-dehydrocholesterol is converted to pre-Vitamin D_3_ by a narrow band of solar ultraviolet radiation (290–320 nm) that undergoes isomerisation in a temperature-dependent manner to Vitamin D_3_. Ten thousand to 20,000 IU of Vitamin D can be produced in 30 minutes of whole-body exposure to sunlight. This endogenously produced Vitamin D enters the blood and binds with Vitamin D binding protein (DBP), facilitating transportation to the liver [1, 5]. Vitamin D can also be obtained from a limited number of dietary sources. Vitamin D_2_ (ergocalciferol) is produced by some kinds of plants and animals when ergosterol is altered by ultraviolet irradiation. However, few foods have naturally occurring substantial Vitamin D_2_ levels to make this a significant source of Vitamin D in humans.

Vitamin D from the skin and diet is hydroxylated in the liver by one of several cytochrome P450 enzymes to the prehormone 25-hydroxy Vitamin D [calcidiol, or 25(OH)D] that is encoded by the gene CYP27A1 [2, 5, 9]. Most circulating 25(OH)D and the active form of Vitamin D, namely, 1,25-dihydroxyvitamin D [1,25(OH)_2_D], are transported in the blood bound to Vitamin D binding protein (DBP) (80–90%) and to albumin (10–20%); only a small fraction remains free or unbound [10]. The 25(OH)D can be taken up by one of two mechanisms, namely, diffusion of free 25(OH)D across cell membranes throughout the body or a DBP receptor mediated endocytosis by the coreceptors megalin and cubulin [5]. It appears that endocytic transportation may be influenced by certain pathological and physiological determinants [11–13]. The amount of DBP and its effect on free versus bound 25(OH)D could inversely influence the available free 25(OH)D for uptake [5]. The primary determinant of how long a Vitamin D metabolite will stay in circulation is its affinity for DBP [14]. Further research is needed to understand the regulation of mobilization of 25(OH)D from lipid storage pools relative to health outcomes.

However, Vitamin D can also be hydroxylated to 25(OH)D in all tissues of the body, achieving autocrine production of 25(OH)D in these tissues [14]. 25(OH)D is then further metabolised in the kidneys and possibly in a wide variety of extrarenal tissues by the enzyme 25-hydroxyvitamin D-1*α*-hydroxylase (CYP27B1) to its active form, namely, 1,25-dihydroxyvitamin D [1,25(OH)_2_D_3_]. Vitamin D exerts its effects by modulating gene expression after binding to VDR. There appear to be potential genetic polymorphisms in key genes with Vitamin D exposure that can influence bioavailability, transport, and distribution in lipid storage pools, metabolism, and action of Vitamin D [5].

## 3. Pain

Pain is part of the human condition. Pain is defined by the International Association for the Study of Pain (IASP) as “an unpleasant sensory and emotional experience associated with actual or potential tissue damage, or described by the patient in terms of such damage” [15]. Pain is essentially a subjective perceptual experience. Pain originates in the nociceptors of the nervous system but the experience of pain is perceived in the conscious brain. It is influenced by a complex interaction of behavioural, environmental, biological, and societal factors.

There are two broad categories of pain, namely, acute nociceptive pain which acts as an early warning sign and pathological persistent pain, which is essentially an ongoing false alarm [16, 17].

Acute pain is a sensation that is elicited after strong stimulation of tissues in the body. Nociception relates to the biochemical and neural changes that occur in response to a noxious stimulus [18]. This stimulation causes action potentials in primary sensory neurones known as nociceptors. These nociceptors are activated by high-threshold stimuli (mechanical, thermal, chemical, or electrical) to transmit excitatory signals to the sensory cell bodies in the dorsal root ganglion (DRG) along the dorsal roots and subsequently into the spinal cord [16, 19]. In the spinal cord, these primary sensory nerve fibres release neurotransmitters such as amino acids (glutamate) and neuropeptides (such as substance P and calcitonin gene related peptide) that activate second-order neurones. The second-order neurones relay information via specific tracts that reach the thalamus where the sensation of pain occurs. Third-order neurones connecting the thalamus to the somatosensory cortex are activated, resulting in the perception of pain [16, 20].

Nociceptors are present in most tissues in the body, including skin, muscle, joints, and viscera. There are predominantly two types of nociceptors involved in the pain pathway, namely, C fibres and A-delta fibres. The thin, myelinated A-delta fibres are fast transmitting; they are activated by both mechanical and thermal stimuli [18]. The unmyelinated C fibres respond to chemical, thermal, and mechanical stimuli. C fibres in the viscera are notable in that they respond to noxious stimuli such as the stretching of hollow organs.

The International Association for the Study of Pain (IASP) defines chronic pain as “pain that has persisted beyond normal tissue healing time.” This is taken (in absence of other criteria) to be 3 months [21]. However, some chronic pain disorders are characterised by recurrent short acute episodes and exacerbations such as trigeminal neuralgia and rheumatoid arthritis.

Chronic pain can be produced after tissue damage (or inflammation), after nerve damage, and after alteration of normal neural function. Chronic persistent pain leads to chemical, functional, and anatomical changes throughout the nervous system (in the periphery, spinal cord, and brain) [22–24]. The concept of neuroplasticity (the ability of the brain to change its structure and function) can be a positive adaptation to loss of function; in the case of pain, it appears maladaptive [25]. Persistent pain alters the nervous system to produce spontaneous pain that arises without any apparent peripheral stimulus as well as a hypersensitivity to peripheral stimuli [16, 17, 26]. Pain hypersensitivity can result in hyperalgesia (the exaggerated and prolonged response to noxious stimulation) and allodynia (pain resulting from a stimulus that is normally nonpainful) [27]. The reduced descending inhibition in the central nervous system (CNS) results in more peripheral noxious signals getting through to the brain resulting in an increased experience of pain [28].

The transition from acute to chronic pain is not well understood and appears to be multifactorial [29]. Nociceptive pain usually (but not always) reduces with healing. While persistent pain is often due to obvious nerve damage (e.g., injury or cancer), there are many instances when no physical pathology can be objectively verified, such as in fibromyalgia syndrome and in headaches. In general, chronic pain may involve a combination of nociceptive and neuropathic mechanisms, as well as some form of central sensitization and learnt response. The concept of central sensitization is becoming more widely recognised, whereby changes occurring within the nervous system result in previously nonnoxious stimuli being perceived as painful, a lowering of the threshold for pain generation, and an increase in the duration, amplitude, and spatial distribution of pain [23]. In addition, central sensitization initiates the interrelationship between many pain problems such as fibromyalgia, irritable bowel, low back pain, and chronic daily headache [30]. Inflammatory and nerve injury are involved in the aetiology of many chronic pain syndromes like osteoarthritis, diabetic neuropathy, or postherpetic neuralgia, but only a small proportion of those subjected to such injuries actually develop chronic pain, and the degree of pain severity can vary significantly between patients [31].

Evidence from large scale studies in Europe, North America, and Australasia has shown that about one in five of the adult population experiences chronic moderate to severe pain with other estimates indicating the prevalence of chronic pain to be closer to 20–25% [32–34]. The incidence of chronic pain can be higher in at-risk groups such as the elderly and the immune-compromised [35, 36]. The rate of persistent severe pain among all residents of United States nursing homes in 1999 was found to be 14.7%, with 41.2% of residents in pain at first assessment experiencing severe pain 60 to 180 days later [37].

The prevalence of chronic pain is projected to increase as the population ages (from around 3.2 million Australians in 2007 to 5.0 million by 2050) [38]. Life-style changes leading to obesity and inactivity will also contribute to increased level of pain in developed countries.

## 4. Interfaces of Pain and Vitamin D

Clinical research in the area of chronic pain and Vitamin D deficiency remains limited. There is a dearth of large double blind randomised controlled studies. However, there is enough evidence showing the potential of Vitamin D to exert anatomic and physiological influences on pain manifestation, thereby playing a role in the aetiology and maintenance of chronic pain states and associated comorbidity [1, 6]. Persistent pain is associated with Vitamin D-related bone demineralization, myopathy, and musculoskeletal pain. Pain pathways associated with cortical, immunological, hormonal, and neuronal changes are potentially also influenced by Vitamin D levels.

Vitamin D levels have been found to be low in certain groups of patients with various pain states (Box 1) [39–43]. Studies of Vitamin D supplementation in patients with known Vitamin D deficiency have shown mixed results in chronic pain patients regarding improved pain scores (Box 1) [6, 9, 44–51]. The prevalence of a variety of pain states at particular latitudes has been linked to low levels of Vitamin D [52–54]. Seasonal variations correspond with varying pain levels as well [6]. Vitamin D deficiency has been associated with headache, abdominal, knee, and back pain, persistent musculoskeletal pain, costochondritic chest pain, and failed back syndrome and with fibromyalgia [6, 45, 53–59].

Long-term Vitamin D deficiencies have been linked to a weakened immune system and to chronic inflammation [2–4, 60]. Chronic inflammation, in turn, leads to debilitating health conditions; many of these are characterised by pain as the disabling symptom [60]. Serum Vitamin D deficiency [25(OH)D] is considered a risk factor for type 1 diabetes, multiple sclerosis, and especially autoimmune rheumatic diseases (ARD) (Box 1). The severity of systemic lupus erythematosus and rheumatoid arthritis has been associated with serum Vitamin D deficiency [61, 62].

Vitamin D deficiency has been linked to other diseases that present with pain as a symptom. Cystic fibrosis patients experience chronic pain in a variety of sites (head, sinuses, back, and chest) [63]. Individuals with cystic fibrosis are at risk of Vitamin D deficiency due to limited sun exposure and malabsorption. Low bone density and osteopaenia appear to contribute to chronic pain in cystic fibrosis patients and are potentially related to low 25(OH)_2_D_3_ levels [63, 64].

Gender differences may be related to Vitamin D deficiency associated with chronic pain. In one large study, prevalence of chronic widespread pain varied by 25(OH)D concentration in women but not in men [40]. Racial differences in experimental pain are mediated by differences in the Vitamin D levels [65]. Vitamin D deficiency may be a risk factor for increased knee osteoarthritis pain in black Americans.

Individuals suffering from chronic pain usually experience other comorbidities such as sleep, anxiety, and mood disorders. These conditions can impact on the patient's quality of life to a significant extent, resulting in loss of employment and/or withdrawal from social life. Adequate levels of Vitamin D have been associated with improved quality of life indicators [48, 66–68]. Significant improvements in assessment of sleep, pain levels, well-being, and various aspects of quality of life with Vitamin D supplementation have been shown [48, 66–69].

### 4.1. Pain Associated with Vitamin D-Related Bone Demineralization

In the absence of bone mineralisation due to Vitamin D deficiency, weight-bearing growing bones (arms and legs) of infants and children become bowed. In infants, rickets may result in delayed closing of the fontanelles and in rib cage deformities [3]. Subtle evidence of rickets in children includes leg pain, delayed age for standing and walking, and delayed growth.

Adult bones undergo a constant state of remodeling. Decreased levels of 25(OH)D facilitate osteoclast genesis with consequent increased bone resorption [9]. Inadequate mineralisation of the collagen matrix due to low calcium and phosphate levels results in osteomalacia. Bone pain and proximal muscle weakness with gait instability are characteristic of osteomalacia [3, 9]. Decreased levels of 25(OH)D have been shown to be directly related to low bone density in the hip [9, 70].

### 4.2. Pain Associated with Vitamin D Deficiency and Muscle Weakness

Vitamin D deficiency causes muscle weakness and pain in children and adults. Individuals with chronic low back pain have been found to have weaker gluteus medius muscles than control subjects without back pain [71]. The incidence of low back pain is associated with isometric and isokinetic trunk extensor weakness, whereas low back pain severity is associated with isokinetic trunk extensor and flexor weakness and isometric trunk extensor and flexor weakness [72].

Vitamin D has an important role in the regulation of serum calcium concentration and in muscle protein synthesis. Vitamin D increases the serum calcium level that is essential for muscle contraction; protein synthesis affects muscle growth [73]. This process is mediated by the nuclear vitamin D receptor (VDR) and by a variety of nongenomic effects [74]. VDRs are found in muscle tissue. Vitamin D improves musculoskeletal function by exerting a direct effect on the muscle tissue itself [75, 76]. Vitamin D deficiency primarily affects the faster and stronger type 2 muscle fibres. This may explain why supplementation with Vitamin D improves proximal muscle strength. Hypovitaminosis D impairs neuromuscular coordination, as measured by body sway. It increases the risk of falling and painful fractures related to falls in the elderly [73, 74, 77–80]. It has been postulated that there is a genetic predisposition to the decline in strength in elderly women that is linked to the VDR [81].

Musculoskeletal pain related to osteomalacia may probably be due to spongy matrix formation under the periosteal membranes caused by demineralization of the bone [82]. This gelatin-like collagen matrix can expand when it becomes hydrated; it causes outward pressure on periosteal tissues that are richly innervated with sensory pain fibres. This pressure results in a throbbing, aching bone pain [82].

### 4.3. Mechanisms of Action of Vitamin D on Pain Processes

There are a number of mechanisms involved in the development of neuropathic pain after peripheral nerve damage (Box 2). These include ectopic excitability of sensory neurones, altered gene expression of sensory neurones, and sensitisation of neurones in the dorsal horn of the spinal cord [27]. These mechanisms are influenced by a number of biological and psychosocial factors. Neurotransmitters such as glutamate, substance P, serotonin, and gamma-aminobutyric acid (GABA) as well as glial inflammation are central to the excitatory and inhibitory influences on pain.

Inflammatory pain and neuropathic pain may depend on the action of diverse cytokines and other molecules; these include eicosanoids, endorphins, calcitonin-gene-related peptides (CGRP), and transcription factors. Because steroid compounds (including hormonal steroids, neurosteroids, and synthetic analogues of neuroactive steroids) control the plasticity of the nervous system, these compounds are of particular interest in the modulation of pain [83].

#### 4.3.1. Vitamin D as a Neuroactive Steroid

Vitamin D can modulate neuronal excitability similar to that of other neuroactive steroids (Box 2) [84]. This includes spontaneous regular firing, action potential duration, intrinsic excitability, and sensitivity to neurotransmitters as well as to neurotransmitter receptors such as GABA receptor and N-methyl-D-aspartate (NMDA) receptor [85–87]. Steroid hormones influence the electrical activity of many neurones and effectors by regulating transcription of their ion channels and neurotransmitter receptors, or by modulating the activity of their channels and receptors through second messenger-coupled membrane receptors [85]. Vitamin D as a neuroactive steroid activates a variety of signal transduction systems. These include calcium ion influx, the release of calcium ions from intracellular stores, the modulation of adenylate cyclase, phospholipase C (PLC), protein kinase C, protein kinase D, the mitogen-activated protein (MAP) kinases, and the rapidly accelerated fibrosarcoma (Raf) kinase pathways [88]. As a steroid, Vitamin D modulates brain neurotransmitters (acetylcholine, dopamine, and serotonin) as well [84].

#### 4.3.2. Vitamin D and Neurotrophins

Vitamin D upregulates the synthesis of neurotrophins such as neural growth factor (NGF), neurotrophin 3 (NT3), and glial cell line-derived neurotrophic factor (GDNF), whereas neurotrophin 4 (NT4) is downregulated (Box 2) [89–92]. Through this system, Vitamin D can potentially affect the development, maintenance, and survival of neurones. NGF is a well-established inflammatory mediator. It has direct effects on the sensory nerve endings causing hypersensitivity, amplification of sensory input signals, and enhanced innervation of injured tissue [93–95]. In the diabetic neuropathic pain model, Vitamin D supplementation has been shown to increase NGF production and prevent neurotrophic deficits [91]. There is some evidence that Vitamin D exercises a neuroprotective function by attenuating the effects of glucocorticoids and by modulating neuronal calcium ion homeostasis [96, 97].

VDRs have been localised in neurones and in glial cells [98]. Genes encoding the enzymes involved in the metabolism of Vitamin D are expressed in brain cells [98]. It has been suggested that the local synthesis of 1, 25(OH)_2_D_3_ by microglia could stimulate an antitumour response; this is because 1, 25(OH)_2_D_3_ causes cell death and redifferentiation programs in glioma cells [92].

In other circumstances, microglia contribute to neuropathic pain after peripheral nerve injury [93, 99–101]. Vitamin D plays a fundamental role in astrocyte detoxification pathways, thereby exerting a neuroprotective effect [90]. Vitamin D increases the synthesis of several neurotrophins in astrocytes; these include NGF, NT3, and GDNF and *γ*-glutamyl transpeptidase (another mechanism involved in neuroprotection) [92].

#### 4.3.3. Vitamin D and Prostaglandins

Vitamin D influences prostaglandin action by inhibiting COX-2 expression and by stimulating 15-prostaglandin dehydrogenase (15-PGDH) expression [102]. The enzyme 15-PGDH degrades prostaglandins and inhibits prostaglandin-E2 receptor (PGE2) subtypes and prostaglandin-F2 alpha receptor subtypes (Box 2) [102]. Prostaglandins have a direct effect on sensory neurones by lowering the firing threshold, increasing the number of action potentials elicited by a depolarizing stimulus, and enhancing SP and CGRP release [103]. Prostaglandins mediate neuropathic pain in the spinal cord via PGE2 depolarising wide dynamic range neurones [27].

#### 4.3.4. Vitamin D Effects on Inflammatory Pathways

Vitamin D is known to affect a number of inflammatory pathways associated with the development and persistence of chronic pain (Box 2). Vitamin D upregulates transforming growth factor beta 1 (TGF-*β*1) and interleukin-4 (IL-4) found in astrocytes and microglia [92]. TGF-*β*1 suppresses the activity of various cytokines, namely, interferon-*γ*, TNF-*α*, and various T cells such as interleukin-1 (IL-1) and interleukin-2 (IL-2). It can downregulate the activity of immune cells through suppression of cytokine receptors (such as the IL-2 receptor) [104].

Vitamin D suppresses TNF-*α* and macrophage colony-stimulating factor (M-CSF) in astrocytes and microglia [92]. TNF-*α* has been convincingly implicated at both peripheral and central levels of sensitization [105]. M-CSF is a cytokine that stimulates proliferation, differentiation, and survival of monocytes and macrophages. Macrophages can release many inflammatory mediators, including proinflammatory cytokines, particularly TNF-*α* and interleukin-1-beta (IL-1*β*), NGF, NO (nitric oxide), and prostanoids [93]. By limiting M-CSF, Vitamin D has the potential to inhibit pain pathways.

#### 4.3.5. Vitamin D and Nitric Oxide Synthase

Vitamin D has also been found to inhibit the synthesis of nitric oxide synthase (iNOS), the enzyme that produces nitric oxide (NO), in macrophages that activates microglia and astrocytes at both protein and m-RNA levels (Box 2) [106]. NO is an important neurotransmitter involved in the nociceptive process; in the dorsal horn of the spinal cord, it contributes to the development of central sensitization [107]. Astrocytes play a pivotal role in central nervous system (CNS) detoxification pathways, where glutathione (GSH) is involved in the elimination of nitric oxide. The activity of gamma-glutamyl transpeptidase (gamma-GT), an enzyme of central significance in GSH metabolism, has been shown to be regulated by 1,25-dihydroxyvitamin D_3_ [1,25(OH)_2_D_3_] causes cell death [108]. The inhibition of iNOS by Vitamin D is a potential mechanism for reducing pain and neuronal damage after injury or in diseases such as ischaemia, Parkinson's disease, and acquired immune-deficiency syndrome (AIDS) [92, 93].

#### 4.3.6. Vitamin D and T-Helper Cells

Several immune cell types contribute to peripheral neuropathy and to the development of neuropathic pain. Mast cells are released by injured nerves; they appear to reduce the recruitment of neutrophils and monocytes into the injured nerve, potentially reducing the release of chemokines and other mediators. Vitamin D downregulates neutrophil function [109]. Neutrophils produce various inflammatory factors (lipoxygenase products, nitric oxide, and cytokines) [93]. High levels of neutrophils are released after tissue injury and are linked to the development of symptoms of neuropathic pain [110]. The hyperalgesic actions of NGF appear to be partly dependent on neutrophil accumulation [93]. Vitamin D inhibits T-helper cell overactivity and plays an important role in the prevention of autoimmune diseases (Box 2) [111].

#### 4.3.7. Vitamin D Receptor (VDR) and 1*α*-Hydroxylase

VDR and 1*α*-hydroxylase [the enzyme that converts 25(OH)D by hydroxylation to the active 1,25(OH)_2_D_3_] are found in many areas of the human central nervous system. These include the prefrontal cortex, amygdala, raphe, substantia gelatinosa, cerebellum, hippocampus, cingulate gyrus, substantia nigra, thalamus, and hypothalamus [84]. Both the receptor and the enzyme have been demonstrated in neuronal and glial cells as well [112]. In the rat model, Vitamin D binding protein has been found in axonal projections in the lateral hypothalamus [113]. The presence of VDRs, 1*α*-hydroxylase, and Vitamin D binding protein in the hypothalamus is suggested as the mechanism by which Vitamin D deficiency is implicated in the pathophysiology of various primary headache disorders (Box 2) [52].

## 5. Vitamin D Associated with Pain Comorbidities (Sleep and Depression)

Anatomical and the functional colocalization of central serotonergic, noradrenergic, and dopaminergic systems involved in pain, sleep, Vitamin D, and depression also tend to point to some interconnectivity (Box 3). The enzyme 1 alpha-hydroxylase which converts 25-OHD to the active Vitamin D is present in the hypothalamus, cerebellum, and substantia nigra, areas that are also associated with depression. VDRs are widespread in the human central nervous system, including neurones and glia in many areas of the cingulated cortex and hippocampus, which have been implicated in the pathophysiology of depression [114]. Vitamin D receptors are also present in the anterior and posterior hypothalamus, substantia nigra, midbrain periaqueductal gray (PAG), raphe nuclei, and the nuclei reticularis pontis oralis and caudalis. These same areas play a role in the initiation and maintenance of sleep. Vitamin D's effects on these brain areas may be linked to sleep modulation [115].

There appears to be considerable overlap in the effects of sleep, pain, depression, and Vitamin D on the immune system. Vitamin D could be linked to depression, sleep, and chronic pain by mediating effects of immune cells such as astrocytes and macrophages (Box 3) [90, 92]. Vitamin D directly affects T cell responses inhibiting the production of IL-2, interleukin-17 (IL-17), and interleukin-21 (IL-21) and by stimulating interleukin-4 (IL-4) production [116].

Chronic sleep loss impairs immune function. This results in increases in proinflammatory mediators such as cytokines and chemokines [117, 118]. Cytokines (especially IL-1beta and TNF-*α*) are implicated in the regulation of sleep and modulation of sleep architecture and appear to be involved in circadian regulation of sleep [118–121].

There are suggestions of a bidirectional feedback loop between sleep and cytokine expression [118]. Insufficient sleep quantity may facilitate or exacerbate pain through elevations of interleukin-6 (IL-6) [122]. In disorders where sleep disturbances are common, insufficient sleep quantity itself may establish and maintain its cooccurrence with pain and with increased inflammation [122]. Vitamin D deficiency could contribute to poor quality sleep or symptoms of impaired wakefulness by inducing a relative elevation of circulating TNF-*α* and nuclear factor kappa-light-chain-enhancer of activated B cells (NF*κ*B), both of which can result in subjective feeling of sleepiness [123].

Patients with depressive symptoms and with pain disorders display enhanced cytokine levels. These include IL-6, C-reactive protein (CRP), IL-1B, and TNF-*α* [124]. Major depression may be accompanied by systemic immune activation or an inflammatory response with involvement of phagocytic (monocytes, neutrophils) cells, T cell activation, increased prostaglandin secretion, and increased production of IL-1*β* and IL-6 by peripheral blood mononuclear cells [125]. Cytokines also play a central role in the generation and transmission of pain with increased levels of TNF-*α* and IL-1, IL-2, and IL-6, and decreased levels of IL-4 and IL-10 [126, 127]. Cytokines have been shown to modulate the central metabolism of serotonin and dopamine [128].

IL-1 stimulates the synthesis and/or release of growth hormone releasing hormone, prostaglandin-D_2_, adenosine, and nitric oxide. These are substances that are implicated in regulation of non-REM sleep [117]. Vitamin D has been shown to downregulate these substances [106]. Vitamin D, chronic pain, sleep, and depression are intimately linked to the nervous, endocrine, metabolic, and immune systems of the body and their mediators. These mediators include neurotransmitters, neuropeptides, hormones, and cytokines [117, 119, 129].

There are four key neurotransmitters involved in Vitamin D, pain, and depression, namely, serotonin, noradrenaline, substance P, and dopamine. Serotonin levels are typically low in depressed patients [130, 131]. Serotonin can have pronociceptive or antinociceptive effects, depending on the receptor subtype and location in the central nervous system. Serotonin can be pronociceptive in the periphery, whereas the primary antinociceptive effects of serotonin are thought to occur via receptors located centrally in the descending antinociceptive pathways [132].

Chronic sleep loss has also been shown to have an effect on a variety of neurotransmitters involved in sleep regulation via increase in IL-1 [117]. IL-1 inhibits acetylcholine release and can potentiate or inhibit the effects of glutamate and has been linked to the stimulation of adenosine and monoamine release, thereby playing a role in sleep homeostasis [117, 119]. IL-1 and TNF also interact with the serotonin system to enhance non-REM sleep [133]. IL-1 reduces the firing rate of wake-active serotonergic neurones by potentiating the inhibitory effects of gamma-aminobutyric acid (GABA) [134]. IL-1 in the preoptic area (POA) of the hypothalamus and the adjoining magnocellular basal forebrain (BF) appears to be controlled by corticosteroids released into the blood by the adrenal cortex. Corticosteroid levels depend on the activity of the hypothalamic–pituitary–adrenal axis that is stimulated by the activation of the serotonin pathway [117]. As mentioned previously, IL-1 and TNF have a direct action sensitising nociceptors, enhancing excitatory currents, and reducing inhibitory pain pathways [135].

Dopamine is integral to the regulation of sleep and wake [136, 137]. Dopamine has been shown to moderate motivation and reward-related behaviour that are typically disrupted in depression [138]. Dopamine also appears to have an antinociceptive effect at both spinal and supraspinal levels. It has an important role in descending inhibition of pain pathways [139].

The reduced synthesis of dopamine in the nucleus accumbens during prolonged exposure to unavoidable stress results in the development of persistent hyperalgesia [140]. Low dopamine levels have been associated with painful symptoms related to fibromyalgia as well [139].

It has also been suggested that serotonergic raphe cells involved in alertness become dysregulated during chronic pain and contribute to sleep disruption and sleep loss [136, 141]. Sleep deprivation has been shown in preclinical studies to dysregulate endogenous opioid systems and reduce the analgesic efficacy of mu-opioid receptor agonists, thereby modulating pain systems [142, 143]. Acetylcholine, adenosine, and GABA are all known to modulate sleep and pain [144]. The pontine reticular formation plays a key role in rapid eye movement (REM) sleep generation and may regulate central-pain processing mechanisms [144, 145].

Depressed patients showed reduced cerebrospinal fluid levels of noradrenaline [146]. Noradrenaline primarily has an antinociceptive effect on the alpha-2 adrenoreceptors in the descending pain pathways centrally by reducing the sensitivity of the dorsal horn neurones to noxious stimuli [147]. However, like serotonin, noradrenaline can also have a lesser pronociceptive effect on the periphery in sympathetically mediated pain [148].

Substance P has been shown to be an important neurotransmitter in pain and depression [149]. Raised cerebral spinal fluid (CSF) levels of substance P have been found in patients with depression [124]. Substance P contributes to central sensitization in persistent pain [149, 150]. Since substance P has been found to be to be present in central serotonergic, noradrenergic, and dopaminergic systems, researchers believe that it may be an important modulator in depression and pain [124].

Proinflammatory cytokines potently activate the hypothalamus–pituitary–adrenal axis [151]. This effect is usually attributed to increased production of corticotrophin-releasing factor (CRF) [151]. CRF appears to have an important role in both depression and pain. Raised levels of CRF have been linked to melancholic depression [152]. CRF has also been shown to be antinociceptive in the central nervous system as well as in the periphery [153]. A hyperactive hypothalamus–pituitary–adrenal (HPA) axis is often associated with clinical depression [151]. Vitamin D modulates this axis by regulating adrenaline, noradrenaline, and dopamine production through VDRs in the adrenal cortex and protects against the depletion of dopamine and serotonin centrally.

## 6. Conclusion

In summary, low Vitamin D levels are implicated in various chronic pain conditions. Research has shown that Vitamin D exerts anatomic, hormonal, neurological, and immunological influences on pain manifestation, thereby playing a role in the aetiology and maintenance of chronic pain states and associated comorbidity [1, 6–8]. More research is necessary to determine whether Vitamin D is useful in the treatment of various pain conditions and whether or not the effect is limited to patients who are deficient in Vitamin D [43].

## Figures and Tables

**Box 1:**
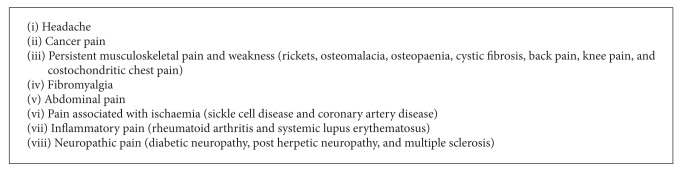
Interfaces of Vitamin D deficiency and types of persistent pain.

**Box 2:**
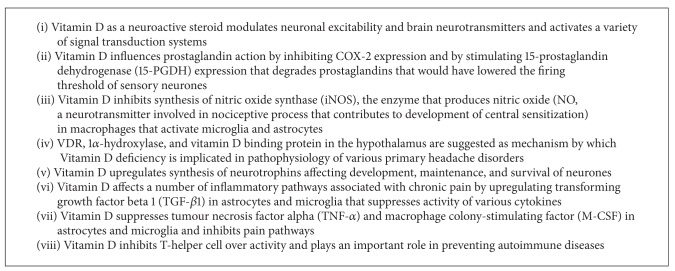
Mechanisms of action of Vitamin D on pain processes.

**Box 3:**
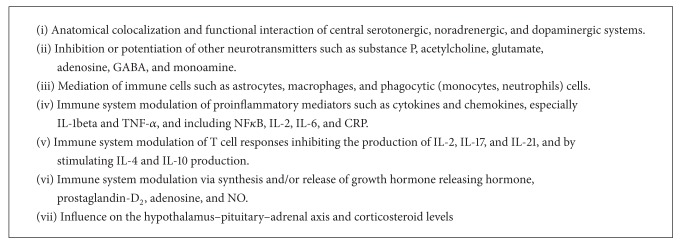
Potential interfaces of Vitamin D, pain, sleep, and depression.
